# Mental health issues is common, resiliency is rare: Qataris and residents’ experiences with distress, traumatic symptoms, and coping during the blockade

**DOI:** 10.1371/journal.pgph.0001804

**Published:** 2023-04-19

**Authors:** Wahiba Abu-Ras, Maryam Burghul, Eliza Decker

**Affiliations:** 1 Adelphi School of Social Work, Garden City, NY, United States of America; 2 Doha Institute for Graduate Studies, Doha, Qatar; ESIC Medical College & PGIMSR, INDIA

## Abstract

Many people feel vulnerable and uncertain about their future during a political crisis or political instability. Nonetheless, people may choose different coping strategies, making some more resilient and others more vulnerable to mental health issues. Added to the stress caused by these political factors is that social media has become the only source of information, including intolerance, hate speech, and bigotry. Therefore, reactive strategies to traumatic events and resiliency are essential components in addressing the affected population’s stress and mental health issues. Although much attention has been paid to the political blockade imposed on Qatar in 2017, little emphasis has been placed on its impact on the affected people’s mental health, coping strategies, and resiliency. The study explores Qatari citizens’ mental health, resilience, distress, traumatic symptoms, and coping in the context of the blockade. This study fills the knowledge gap in this area by using a mixed-method approach with 443 online surveys and 23 face-to-face interviews. Quantitative data shows women scored higher on distress than men (17.37 v 9.13, p = .009), but men scored higher on resiliency (73.63 v 68.19, p = .009). Qualitative data supported these findings. The findings will lay the foundation for clinical trials and social interventions to provide better mental health services to Qatari families directly affected by the blockade and inform mental health providers and policymakers about stress, coping strategies, and resilience during this crisis.

## Introduction

Political instability is likely to affect every aspect of people’s lives. The short-term thinking of policymakers can also cause them to make suboptimal macroeconomic decisions. A frequent policy change may lead to instability and negatively affect macroeconomic performance [1]. Furthermore, political instability can undermine people’s sense of belonging, safety, mental health, and well-being [2]. Studies suggest that psychological and physical health are adversely affected by political violence, social differences, and economic disparities [3,4]. Social disadvantage and bias also affect mental health, knowledge, and behavior about health and access to services. World Health Organization [5] estimates that 22% of people living in conflicted areas suffer from mental disorders. Approximately 13% of participants suffer from mild depression, anxiety, and post-traumatic stress disorder, while 4% suffer from moderate forms [3]. The literature in this field also indicates that family separation, such as that caused by the blockade, can have devastating short- and long-term traumatic effects on the most vulnerable members of society, including children and their parents [4–8].

There are several major humanitarian crises in the Middle East and North Africa (MENA), including those in Iraq, Libya, Palestine, South Sudan, Syria, Western Sahara, and Yemen. Some of these crises lasted for decades, and others just emerged. Recently, there has been a political conflict involving several countries in the Gulf region. Bahrain, United Arab Emirates, Saudi Arabia, and Egypt imposed a sea, land, and air blockade on Qatar in 2017. For over three years, these Gulf countries continued to exert pressure on Qatar at various levels, positively and negatively affecting Qatar’s political, economic, and social systems and its citizens’ well-being. In particular, cross-national families and children have been affected, suffering severe human rights violations that have damaged family unity and cohesion [6]. Although the Gulf crisis has received considerable attention from national and international news media (Twitter and Facebook), few empirical studies have been conducted to address the severe impact this crisis has had on Qatari citizens and residents. This study will address the consequences of the blockade imposed on Qatar and explore how Qatari citizens and residents reacted to the event, their coping strategies, and resilience.

Using the Kuwaiti Invasion, [7] the stress, deprivation, and other issues faced by citizens under the sanctioned countries represent violations of economic, social, cultural human rights [8]. Qatar’s high standard of living and excellent employment opportunities have brought together people from all over the Gulf region. The ease of traveling back and forth and the incentives of being a Qatar national resulted in various Gulf families settling in the state of Qatar; these families considered Qatar their second home. However, the blockade may have taken a heavy toll on this familial solidarity between the Gulf nations, as it coerced the separation of family members.

The joint statement issued by the blockading countries intentionally focused on destroying the social fabric of the Gulf region [9], breaking the Gulf families with Qatari members into fragments. Due to the absence of a national parent in a blockading country, children were separated from one of their parents [10]. This situation even led to divorces within cross-national families [11]. As a result, many families distanced themselves from their Qatari relatives, fearing jail sentences and heavy fines if they sympathized with Qatari nationals [12,13]. Also, the spread of misinformation and disinformation among Gulf cross-national families increased fear and anxiety [14] and destabilized family units. The hostile climate in the region continued to affect separated families even as they tried to reunite with each other on social media [15].

Qatar had to face an international social media trial, with no control over what was being shared; this situation resulted in increased misunderstandings, rifts, anxieties, and suspicions between relatives of these families [16]. Most available information on social media reported negative sentiments and described this event as “unforgettable,” “unforgivable,” and “painful” [17]. Another study [18], analyzing Twitter messages, concluded that the blockade has been traumatic for Qatari citizens and that residents of Qatar reported phrases similar to those used by mental health patients who suffer from depressive symptoms or pessimistic thoughts. These tweets also formed thematic patterns related to abuse, fear, and anxiety related to psychological factors. However, the tweets did not address the blockade’s impact on Qatari and their families, challenges with family separation, coping strategies, or resiliency. Another study conducted by the Doha International Family Institute (DIFI) [19] on the overall impact of the blockade on Qatari families used a face-to-face interview with 22 individuals and two focus groups. DIFI study showed that many families reported legal, economic, social, and psychological challenges, making them more vulnerable to collective and individual trauma and psychological stresses. Most studies, however, focused on the Gulf crisis coverage and reactions. For example, studies [20,21] discuss public officials and the role of media discourse regarding the blockade. The Social and Economic Survey Research Institute [22] used phone calls with 889 Qatari citizens to gauge participants ‘perceptions of the blockade’s political, legal, social, and economic implications on Qatari society. Two other studies addressed the political [23] and financial implications for the region [24] and the relationship with the US national interest [25]. There is no comprehensive study on the impact of the psychological effect of the blockade on families, coping strategies, and resiliency for this target population. This study is one of the pioneers, if not the first, to use a mixed-method approach and comprehensive measures and scales to assess the psychological impact of the blockade on Qatari citizens’ and residents’ well-being, coping strategies, and resiliency.

### Mental health in Qatar: Overview and significance

The availability of mental health services is still limited in Qatar. According to the National Health Strategy (2018–2022) report: “Qatar has fewer outpatient facilities, beds in community residential facilities and beds in psychiatric hospitals” [26]. However, services continue to develop across primary care, including the development of mental health support clinics offering psychological interventions at par with other high-income countries. The need for these professionals is crucial because there was a significant increase between 2012 and 2014 in the average number of contacts per user in mental health outpatient facilities from 3.49 to 4.38. Outpatient female users were close to 50% in 2014, and there was a 7% increase (23%) of children and adolescents [27]. Based on this increase, the Ministry of Public Health in Qatar began to develop a strategy to address mental health illness by reforming the mental health system in the country. An example is the National Mental Health Strategy for Qatar, “Changing Minds, Changing Lives 2013–18” [27], which supports the transition of the mental health system towards more community-based care [26].

Despite the changes and improvements in this field, Qatar severely needs more mental health professionals compared to high-income countries. This includes psychiatrists, nurses, social workers, psychologists, and occupational therapists. In 2014 there were 13.5 mental health professionals per 100,000 people, less than half the median rate of upper-middle-income countries [26]. The provision included 3.04 psychiatrists per 100 000 population, 8.19 nurses, 0.89 psychologists, 0.50 occupational therapists, and 0.36 social workers. In 2014, the total national expenditure on mental health was 0.36% of all healthcare expenditure, nearly seven times lower than the median for upper-middle-income countries, and mainly directed towards in-patient services in the mental hospital [26].

The status is even more alarming considering the low number of professionals acquiring training in mental health care in Qatar’s social, medical, and educational institutions [28]. They are, therefore, inadequately trained to respond to complex mental illnesses such as depression, trauma, crisis intervention, or couple therapy.

### Reaction to trauma, coping strategies, and resilience

The American Psychological Association [29] defines resilience as “the process of adapting well in the face of adversity, trauma, tragedy, threats, or significant sources of stress -such as family and relationship problems, serious health problems, or workplace and financial stressors.” In human capability, resilience is the ability to recover from adverse events using positive emotions. A resilient mindset can be cultivated after facing significant challenges and difficulties when adapting to new unfavorable situations [30]. There are two main axes along which resilience develops throughout life: an intrapsychic axis related to an individual’s capabilities and a relational axis related to human relationships with others (parents, family, teachers, communities, etc.) [31]. Resilience can vary greatly depending on an individual’s specific circumstances and challenges, and each person can behave differently in response to the same event. Trauma and adversity do not always lead to impairment and mental health problems; some individuals recover and develop resilience.

Recent research suggests that the emotional burden carried by trauma-exposed adults and children in the Middle East is substantial, with high Post Traumatic Stress Disorder (PTSD) rates that can be debilitating for those who are an undiagnosed and untreated large population [32]. Culture and religion play a significant role in resilience [33]. Also, coping strategies and resilient practices vary from one region, culture, and religion, with systemic contexts impacting traditional perspectives and practices of resilience [34]. In Qatar, religion plays a significant role in well-being. Religion-based coping refers to using religion-based methods of dealing with stressful situations [35]. It can reduce depressive symptoms, which is why it is a valuable coping strategy for the elderly.

Resilience may take on a different meaning during a political conflict, and there are numerous ways to cope with stress, each of which has its effects. Micro-level resilience emphasizes the ability of individuals to cope with difficult or traumatic circumstances [36]. Mezzo-level resilience involves overcoming productivity challenges and being prepared to serve effectively (e.g., business communities, transportation, education, housing, employment, law enforcement, health, and human services) [37]. Macro-level (e.g., government) resilience means the ability to anticipate, absorb, accommodate, or recover from hazards promptly and efficiently, including maintaining, restoring, or improving its basic structures and functions [38].

### Theoretical Framework: The ABC-X Model

**Fig 1:**
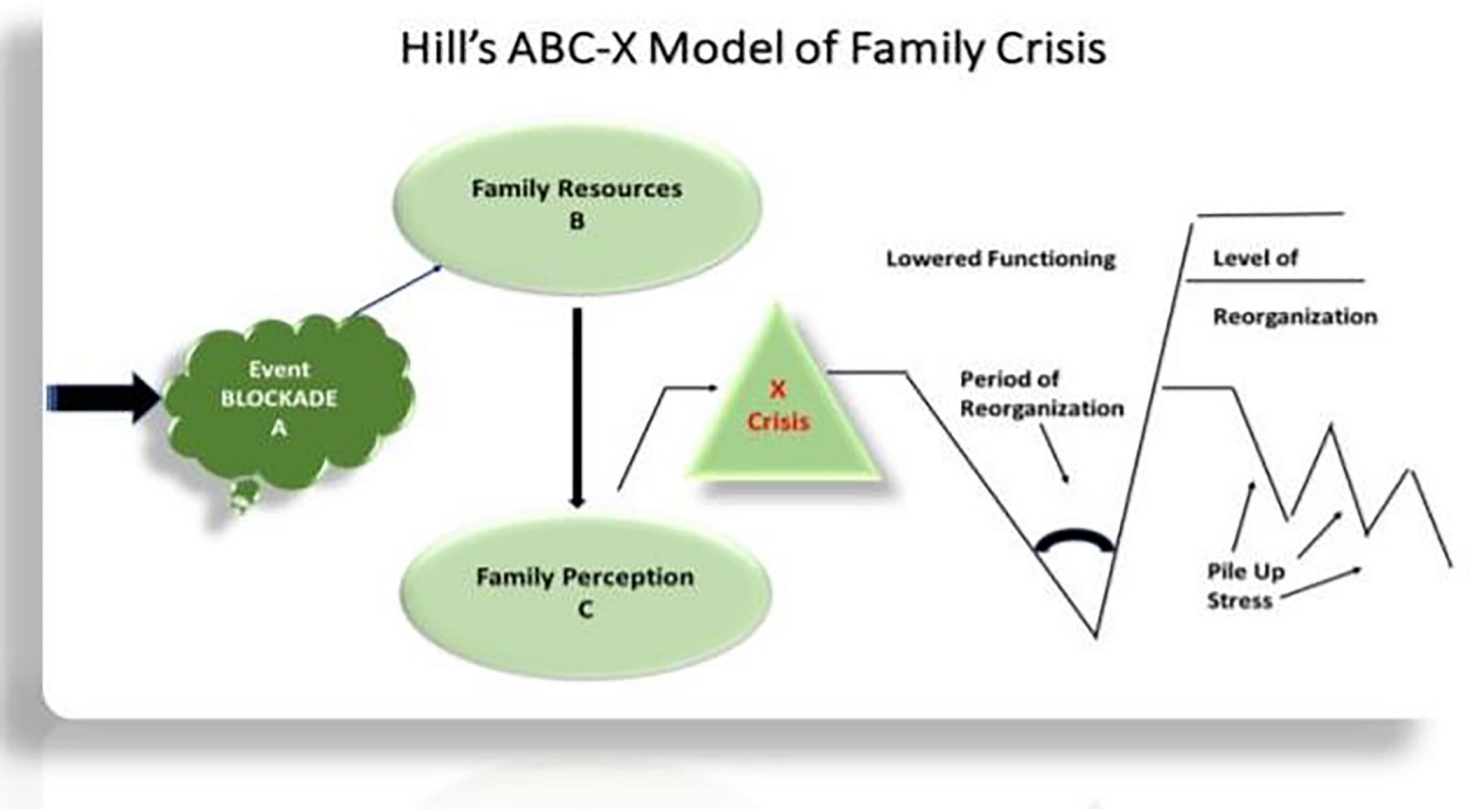
Hill’s ABC-X Model of Family Crisis. Event (A) filters through Family Resources (B) and Family Perception (C) to determine the experience of the Crisis (X). Following the crisis, a period of reorganization occurs at the lowest point of functioning, then level of reorganization rises with level of functioning, while pile up stress occurs in the process.

The ABC-X model guides this study (Fig 1). The ABC-X model [39] assumes that to understand whether an event in the family system (separation/deportation) becomes a crisis (X) (psychological distress), we need to understand three other factors: (A) the stressor related to this particular event (the Blockade), (B) the family’s resources (access to formal and informal support services and parents’ characteristics and resiliency), and (C) the individual’s and family’s reactions to and perception of the event. The main idea is that the stressors’ impact on the family’s psychological health and (X) their psychological distress is influenced by other factors, such as the available internal and external resources such as access to services or personal resources (family perception, coping skills, and resiliency) and their views on the situation. Stressors include positive and negative changes in a family’s social norms and context, such as a job change entails an increase in financial and time resources. Family members, relationships within the family, or the family system can experience stress from any new situation or event that requires substantial adjustments. Due to the blockade, individuals may feel high stress due to disconnection from extended family members or children, especially in cross-national families.

## Methodology

### Ethics statement

This research project was approved on ethical grounds by Adelphi University on 13 February 2020 (BEH1661). Informed consent (in writing and verbally) was obtained from all the participants except for three, who audio-recorded their verbal consent before the interviews.

### Methods

This study used mixed methods of quantitative (cross-sectional survey) and qualitative (individual semi-structured interviews). This design sought to draw upon the strengths and minimize the weaknesses of quantitative and qualitative data in a single study [40], to provide a more inclusive understanding of research problems. The data was collected between November 2019- December 2021 with some interruption in between due to the breakout of COVID-19.

The aims of the study were to (1) explore the psychosocial effects of the blockade on families in Qatar with an emphasis on cross-national families using both quantitative and qualitative approaches; (2) explore major stressors, barriers, risks, challenges, and coping strategies used to suggest ways to improve families’ quality of life, and (3) assess the relationship between psychological stressors and resilience of the target population.

### Quantitative data

#### Sample size

Using the statistical software G*Power, a multiple regression analysis will require the largest sample size. A sample size of at least 172 subjects is required to achieve a power of .80 given an alpha of .05, a medium effect size of .10 with ten independent variables (factors) in the analysis. Given the study’s sensitivity and perhaps a response rate as low as 50%, we aimed to recruit at least 250 participants. However, our sample size reached to 437 completed surveys.

The sample size was based upon the need to recruit adequate numbers to allow explorations of associations within Qatari and cross-national families as opposed for adequate power to test a priori hypotheses. For that reason, we opened recruitment during a set time frame. Similar to other web-based surveys approximately half the respondents did not complete the survey.

#### Recruitment and eligibility

Recruitment for the quantitative study relied primarily on community-based organizations and networks as they already had a rapport with the residents, who would then be more likely to complete such a web-based survey on sensitive issues. The potential participants were linked to the website, where they were informed about the purpose and confidentiality and were then invited to participate in the 15-minute survey. At the end of the survey, the participant received a link to an external site where they could enter to win up to $50 in gift cards as compensation. There was also information about the possibility of participating in individual interviews. The sample size was based upon the need to recruit adequate numbers to allow explorations of associations within Qatari and cross-national families as opposed to adequate power to test a priori hypotheses. For that reason, we opened recruitment during a set time frame.

Participants were eligible to take the survey if they were Qatari citizens (national) or a resident of Qatar, currently residing in Qatar, and at least 18 years and older.

#### Measures

The two measures of mental health in the survey were the Kessler Psychological Distress Scale (K-5**)** [41] and the Impact of Event Scale-Revised (IES-R) [42]. The K-5 measures psychological distress with five questions with responses ranging from “1 = none of the time” to “5 = all of the time”. An example is, “*In the last four weeks*, *about how often did you feel nervous*.” It is widely used to measure psychological distress across cultures and ethnicities [43,44]. The scale has been translated into Arabic with high reliability and validity [45]. The total score ranges from 5 to 25, with a higher score indicating more distress [46–48].

The Impact of Event Scale-Revised (IES-R) [42] measures the presence and severity of traumatic symptoms in the past 7 days. It has 22 self-reported items using a 5-point Likert scale (0 = not at all to 4 = extremely) with scores ranging from 0 to 80 (although means can be calculated) and higher scores indicating greater traumatic symptoms. An example of an item is *“Any reminders brought back feelings about it*.*”* The scale has a high alpha reliability coefficient (0.92) in a similar population [49]. As the scale is based on the criteria used for diagnosing PTSD, subscales of Intrusion, Avoidance, and Hyperarousal can be calculated. Means are recommended for the subscales to allow comparison with scores from the Symptom Checklist 90—Revised (SCL-90-R)

Resiliency was measured with the Connor–Davidson Resilience Scale (CD-RISC) [50]. The CD-RISC scale is a 25-item scale with each item having a 5-point Likert scale ranging from 0 = “not true at all” to 4 =, “true nearly all the time.” Examples are “*Able to adjust to change” and “Pride of your achievement*.*”* The total score ranges from 0 to 100, with higher scores reflecting greater resilience and stress-coping ability. The scale has a high alpha reliability coefficient in published studies (0.89).

A modified version of the Perceived Experience Effects Scale (24-item scale) (PIPES) [51] used to measure the experiences of Qatari families and their children with the effects of the blockade and its consequence policies. The scale was translated into Arabic by two native Qatari translators and two bilingual translators. The items reflect parents’ experiences following the blockade and the implementation of restrictive mobility in the region. Questions address issues related to their experiences with discrimination (e.g., *Were you treated unfairly because of your nationality*?*)*, social exclusion (*e*.*g*., *Did you avoid certain locations like parks and neighborhoods because you did not feel safe*?*)*, a threat to family (*Did you worry about family separation due to deportation*?*)*, children vulnerability (e.g., *Have you been concerned that your children were having emotional problems due to the blockade policies*?*)*, and family (*Did you worry about the impact of these policies on your family*?*)*.

Some examples with service utilization (e.g., *"I saw a doctor or nurse for medical care for my child"; "I met with a lawyer for legal assistance"; "My child attended additional classes at or outside of school"*). Answers to the 24 items include three options:1 = yes, 2 = No. The scale reported Cronbach’s alphas ranged from .82 to .94.

#### Analysis

Prior to analysis, responses were reviewed for completeness, patterns, and outliers. Similar to other studies using web-based surveys, the number of surveys completed on the K-5, IES-R, and CDRS-25 scales (n = 443) was fewer than those that started (n = 729), with the number of missing responses for each question increasing as the survey progressed. Due to this pattern, no attempt was made to impute values for missing data. Of the variables in the analysis, six responses were missing for gender, but all the other variables were complete.

Responses to items within scales were examined for patterns of responses and then scored according to published guidelines. The reliability coefficient for the three scales in this sample was high (0.89 for K-5, 0.86 for IES-R, and 0.93 for CDRS-25). The next step was calculating descriptive statistics to characterize the sample.

As the survey was intended to explore the mental health and resiliency of the people exposed to the blockade, we did not have a priori hypotheses. However, we did examine associations between the mental health and resiliency measures for the sample and subgroups using Pearson correlations, t-tests, and chi-square. These latter explorations are reported as testing for differences by sex and nationality (Qatari vs. other nationalities). The bivariate analysis also examined Qatari nationals for sex (male vs. female) differences and marital status for women.

### Qualitative data

#### Recruitment and eligibility

About 50 people from the survey expressed interest in participating in the individual interviews. We contacted all potential participants using their phone number or email, depending on their preferences indicated in the survey, and 23 only agreed to participate. They were contacted again to confirm the meeting, along with directions to the location and the research team’s contact information. At the meeting, participants were screened again, briefed about the study’s objectives, and asked to read and sign a consent form attached to a letter describing the study’s purpose, potential benefits and risks, confidentiality protection, voluntary participation, and investigators’ contact information. Participants were eligible to take part in this study if a Qatari citizen (national) or a resident of Qatar, a Qatari citizen who was studying in a country that imposed the blockade on Qatari citizens, residing in Qatar, or a spouse of a Qatari citizen and at least 18 years and older.

#### Measures

A semi-structured questionnaire was used with 23 Qatari and non-Qatari cross-national family participants. Each participant provided information via a set of predetermined questions asked the same way regarding their thoughts, opinions, and experiences with psychological distress, perceived support, coping, and resiliency. This approach is ideal for interviewing a large population sample because the responses are easily comparable despite multiple interviewers. Participants were asked about their experiences during the blockade, reactions to the event, and resiliency sources. These questions were included in the survey only.

Other questions were added to address our study’s primary objectives. Each interview lasted between 60–120 minutes. All interviews were conducted by the first author and co-investigators in a quiet and confidential area chosen by the participants. The Principal Investigator is bilingual and lived in the country for two years before the study began. In addition to the first interviewer, a graduate social worker has also been involved in the larger project for several years. As part of the coding process, two additional graduate social workers were hired, one of whom is a Qatari social worker. The principal investigator trained and closely supervised all members of the research team. All interviews except one were audiotaped with a digital recorder and transcribed, then translated from Arabic into English by a professional transcriber and translator. One participant expressed concerns about being recorded, fearing retaliation. Nonetheless, he participated fully in the interview and answered all questions. Immediately following the interview, both interviewers met to discuss and reflect on the interview and compare notes.

#### Analysis

Given our study’s exploratory nature and the lack of relevant data and scientific research on this specific population, we used a grounded theory approach. Grounded theory was used for several reasons. There was little information and knowledge about the nature and impact of the blockade imposed on Qatar and its effect on its citizens’ and residents’ well-being. Information about the blockade was predominantly published in newspapers and social media during the event’s first year. Therefore, it is very appropriate and helpful to use ground theory since it reveals a process inherent in the investigation’s substantive area.

#### Coding process

Coding is a key component of grounded theory methodology. It is the process of conceptualizing and integrating data as theory [40]. There are two types of coding in grounded theory studies: substantive coding, including open coding and selective coding procedures, and theoretical coding. As part of substantive coding, researchers fragment and analyze data directly, first by openly coding, then by sampling and selectively coding data to theoretically saturate the core and related concepts through theoretical sampling and selective coding.

**Fig 2:**
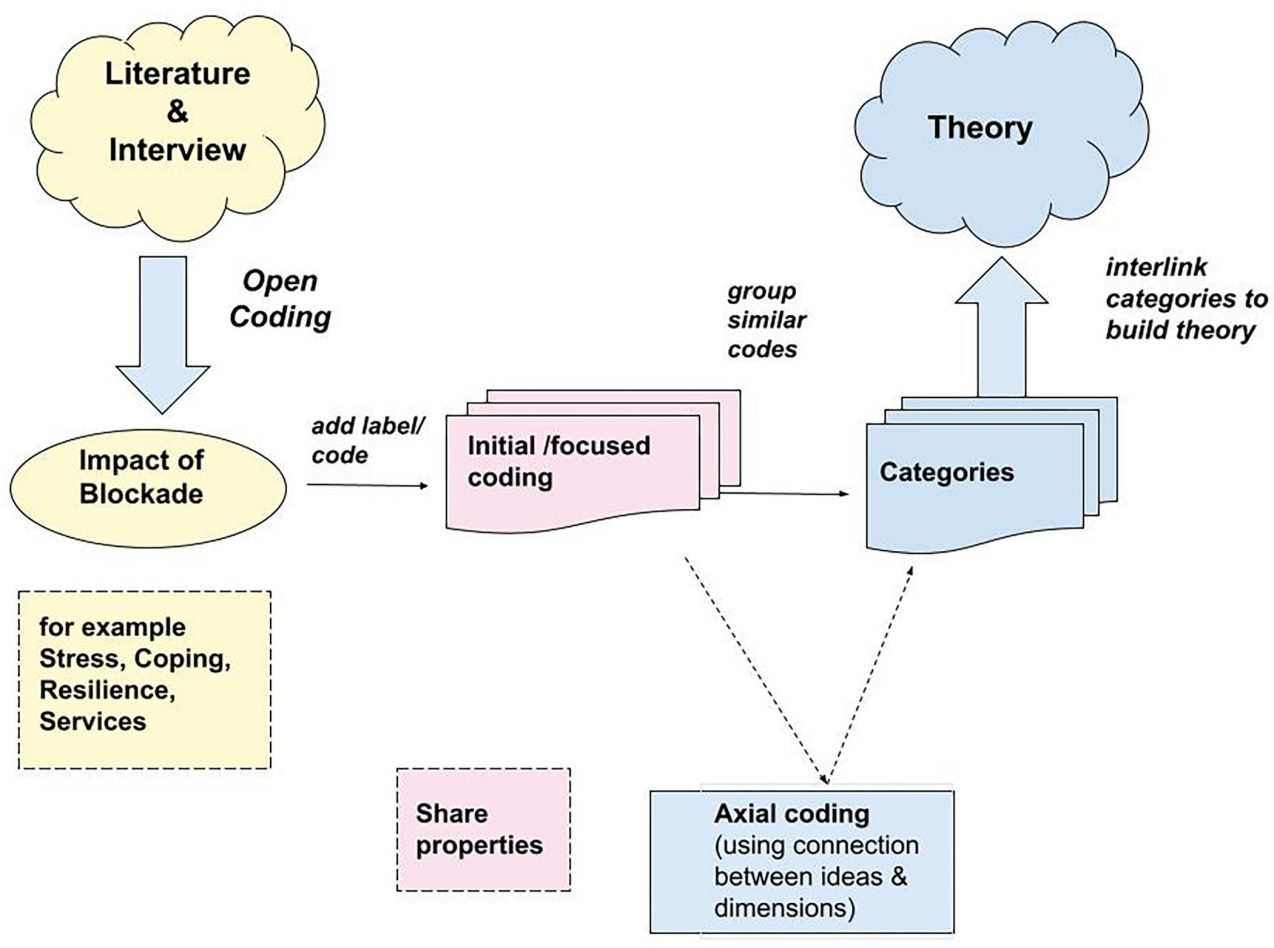
Coding tree. Literature and interviews on the impact of the Blockade are labeled during initial focused coding based on shared properties. Axial coding uses connections between ideas and dimensions groups similar codes and then categories are interlinked to build theory.

As the Coding tree (Fig 2) illustrates, the research team conducted a thorough literature review of the event and its historical and current background. After reviewing the interview transcripts, we followed these steps: First, we collected literature on the blockade using information from newspapers, social media, and Human Rights reports. Then, two research team members and the PI were engaged in the coding process, each having two of the same randomly selected transcripts. Each member assigned meaningful labels/codes to smaller chunks of data, such as sentences and statements. Each selected statement or sentence was highlighted with a specific color, and an explanatory note was made in the margin of each document/transcript. Then each tag or label was connected to a specific context. All team members met to discuss the thematic issues that emerged from each interview, and each presented the list of coding produced. Then coding was compared between all coders. When a disagreement arises among the coders about the exact meaning of a code, we reach out to an outsider who specializes in the coding process, seeking more interpretation and suggestion of labels for those conflicted codes. There was minimum disagreement among the coders because all coders were familiar with the target population’s language and dialect. It is important to note that all coding was data-driven and based on the research questions and answers from the interviewees.

Research team members considered variables of difference (e.g., gender, marital status, nationality, and having children) among participants in assessing the blockade’s influence on their children’s well-being. To articulate contrasts among the categories that emerged, all codes were classified and written separately.

## Results

### Quantitative results

The sample (Table 1) was predominantly female (81.5%) and married (71.6%), with a mean age of 36.47(SD = 9.21). The mean age for women was similar to that of men (36.58 versus 35.99, p = .61). Moreover, there was no gender difference in marital status using the three collapsed categories (Married 72.3% women v 68.0% men; Single 20.8% women v 25.3% men; Other 6.9% v 6.7%, p = .69). Of those married, 92.4% said they had at least one child. There was the expected relationship that single people were the youngest (mean age = 28.65), followed by those married (mean age = 38.04), with the oldest group being the other marital status category (mean age = 44.72), p < .001.

**Table 1:** Sample characteristics. Sample characteristics of the entire sample including demographics, marital status, and mental health symptoms.

Demographics	Total (n = 443)
Qatari nationality, %	63.2%
Age, mean (SD)	36.49 (9.14)
Female, %	81.5%
**Marital Status, %**	** **
Married	71.6%
Single	20.3%
Separated	4.3%
Divorced	1.4%
Widowed	2.55
Have Children (Only asked of those ever married)	67.9%
**Mental Health**	** **
Distress (Kessler 5), mean (SD)	11.01 (4.86)
Traumatic Symptoms (IES-R), mean (SD)	29.95 (14.01)
Intrusion, mean (SD)	1.37 (0.84)
Avoidance, mean (SD)	1.59 (0.85)
Hypervigilance, mean (SD)	1.04 (0.76)
Resiliency, (CDRS-25), mean (SD)	69.44 (16.03)

The score on the K-5 (Table 2) was positively correlated with traumatic symptoms (r = 0.40, p < .001), and negatively correlated with resiliency (r = -0.39, p < .001). The negative correlation reflects that people who reported less stress reported more resiliency. However, the score for the IES-R was not correlated with resiliency (r = -.07, p = .16), suggesting that resiliency helps alleviate general distress but not symptoms following a significant traumatic event. Notably, as age increased, the score on the K-5 decreased, indicating that respondents reported less distress (r = -0.17, p < .001). There was no evidence of multicollinearity among the continuous variables.

**Table 2:** Correlations of mental health scales and age. Correlations between mental health scales and age of participants with significance scores.

	Distress	Traumatic symptoms	Resiliency
Distress (K-5)			
Traumatic Symptoms (IES-R)	0.40**		
Resiliency (CDRS-25)	-0.39**	-0.10*	
Age	-0.17**	0.12*	.08

*p < .05

**p < .001.

In the bivariate analysis (Table 3), women had higher mean K-5 scores than men (11.47 v 9.05, p < .001), but men had higher mean CDRS-25 scores CDRS than women (73.63 v 68.19, p = .009). There were few differences between Qatari and non-Qatari participants (Table 4). Qatari respondents were more likely to be female (87.1% v 71.7%, p < .001) and older (38.26 v 33.45, p < .001). However, there were no differences in the mental health scales by nationality. Within Qatari nationals (Table 5), women had higher distress than men (11.19 v 7.72, p < .001). They also had higher mean scores on the hypervigilant subscale of the IES-R (1.10 v 0.78, p = .024).

**Table 3:** Bivariate analysis by sex (n = 437). Bivariate analysis by sex with sample size 437 (356 female; 81 male) showing prevalence of distress, traumatic symptoms, and resiliency.

Variable	Female (n = 356)	Male (n = 81)
Age	36.57 (9.16)	36.53 (9.10)
Qatari Citizenship**	68.0%	44.4%
Married	71.6%	70.4%
Single	19.7%	23.5%
Other Marital Status	8.7%	6.2%
Distress (Kessler 5), mean (SD)**	11.47 (5.00)	9.05 (3.99)
Traumatic Symptoms (IES-R), mean (SD)	30.35 (13.89)	28.31 (14.79)
Intrusion, mean (SD)	1.38 (0.88)	1.36 (0.88)
Avoidance, mean (SD)	1.61 (0.82)	1.51 (0.96)
Hypervigilance, mean (SD)	1.07(0.77)	0.90 (0.70)
Resiliency, (CDRS-25), mean (SD)*	68.55 (16.03)	73.06 (15.98)

*p < .05

**p < .001.

**Table 4:** Demographic and mental health scales by Qatari citizenship (n = 443). Demographic and mental health scales by Qatari citizenship with sample size 443 (280 Qatari, 163 non-Qatari).

Variable	Qatari (n = 280)	Non-Qatari (n = 163)
Age**	38.26 (9.11)	33.45 (8.39)
Female**	87.1%	71.7%
Marital status		
Married	68.9%	76.1%
Single	20.7%	19.6%
Other marital status	10.4%	4.3%
Distress (Kessler 5), mean (SD)	10.76 (4.81)	11.45 (4.93)
Traumatic Symptoms (IES-R), mean (SD)	30.85 (14.53)	28.40 (12.97)
Intrusion, mean (SD)	1.46 (0.87)	1.22 (0.77)
Avoidance, mean (SD)	1.60 (0.86)	1.58 (0.83)
Hypervigilance, mean (SD)	1.06 (0.80)	1.00 (0.68)
Resiliency, (CDRS-25), mean (SD)	69.37 (16.19)	69.55 (15.82)

*p < .05

**p < .001.

**Table 5:** Demographic and mental health scale differences within qatari by sex (n = 278). Demographic and mental health scale differences within Qatari citizens by sex with sample size 278 (242 female, 36 male).

Variable	Female (n = 242)	Male (n = 36)
Age	36.57 (9.16)	36.53 (9.10)
Marital status	38.46 (9.05)	36.94 (9.63)
Married	61.1%	69.8%
Single	27.8%	19.8%
Other marital status	10.3%	11.1%
Distress (Kessler 5), mean (SD)**	11.19 (4.89)	7.72 (2.99)
Traumatic Symptoms (IES-R), mean (SD)	31.41 (14.50)	27.28 (14.61)
Intrusion, mean (SD)	1.48 (0.87)	1.34 (0.87)
Avoidance, mean (SD)	1.62 (0.83)	1.48 (1.04)
Hypervigilance, mean (SD)*	1.10 (0.82)	0.78 (0.61)
Resiliency, (CDRS-25), mean (SD)	69.12 (15.98)	71.28 (17.85)

*p < .05

**p < .001.

Across the entire sample (Table 6), married women were older, marginally less resilient, and had marginally more severe traumatic symptoms than single women. There was no difference on the other mental health scales. Within the Qatari subsample, married women were older, less resilient, and with more severe traumatic symptoms, especially intrusive ones. These associations were not due to older age, as age was not correlated with resiliency or traumatic symptoms. However, unlike the total sample, resiliency was negatively correlated with traumatic symptoms (r = -.37, p < .001). The lack of correlation between mental health scales and age suggests that married women’s scores were not due to their older age but to marital status.

**Table 6:** Analysis of marital status for women for the entire sample and for Qatari subsample only (n = 278). Analysis of marital status for women for the entire sample and Qatari subsample with sample size 278 showing prevalence of distress, traumatic symptoms, and resiliency.

Entire sample				Qatari subsample		
Variable	Married (n-255)	Single (n = 70)	*p-*value	Married	Single	*p*-value
Age	37.89 (8.42)	29.04 (7.58)	< .001	40.24 (7.84)	29.71 (7.78)	< .001
K-5 (Distress)	11.49 (4.91)	11.46 (4.89)	.97	11.38 (4.99)	10.71 (4.39)	.40
IES-R (traumatic symptoms)	30.91 (13.48)	27.44 (14.08)	.09	32.59 (14.36)	27.60 (13.61)	.033
Intrusion	1.41 (0.83)	1.23 (0.77)	.14	1.57 (0.86)	1.23 (0.80)	.016
Hyper.	1.09 (0.75)	0.98 (0.77)	.27	1.15 (0.82)	0.93 (0.74)	.093
Avoid	1.64 (0.79)	1.47 (0.87)	.14	1.64 (0.80)	1.52 (0.85)	.36
CDRS-25 (Resiliency)	67.38 (16.19)	71.27 (14.50)	.07	67.53 (16.45)	73.21 (12.78)	.028

To explore and tease these associations apart, multivariate regression was conducted with K-5 as the outcome variable. The main effects of being a woman and Qatari nationality were significant (p < .001) even in the presence of CDRS-35 and IES-R scores. Interactions were also explored. The interaction between being a woman and Qatari nationality was not significant (p = .074) in this sample, possibly due to the few male Qatari in the sample. Overall, the model explained 35.1% of the variance in distress.

Women tended to be more likely to report trouble (43.6%) compared to men (31.3%, p = .07) but there was no difference by Qatari citizenship (citizen 42.3% and noncitizen 40.6%, p = .76). There was no gender difference within the subsample of respondents who were Qatari citizens (women 43.6% and men 29.6%, p = .17). However, within the subsample of women who were Qatari citizens, married women were more likely to report trouble (52.0%) than single women (22.6%, p = .003).

As shown in Table 7, the analysis of the relationship between receiving support, seeking help, and experiencing problems revealed that those experiencing more distress and traumatic symptoms were more likely to experience problems, seeking help and support. In addition, respondents without problems reported higher resiliency than those with any problem.

**Table 7:** Relationship between perceived support, help-seeking, and other event related experiences with the blockade and resiliency, traumatic symptoms, and distress. Relationship between perceived support, help-seeking, and other event related experiences with the Blockade and resiliency, traumatic symptoms, and distress.

Variable	Support (n = 208)		*p*-value	Help-seeking (n = 397)		*p*-value	Perceived experiences (n = 334)		*p*-value
	Any (n = 38)	None (n = 170)		Any (n = 115)	None (n = 282)		Any (n = 139)	None (n = 195)	
CDRS-25	64.97 (17.06)	69.53 (15.98)	.118	68.14 (16.96)	70.35 (14.99)	.224 (unequal variances)	67.44 (17.92)	71.98 (14.51)	.014 (unequal variances)
IES-R	35.82 (16.60)	30.17 (13.10)	.055 (unequal variances)	32.30 (13.67)	29.30 (14.00)	.052	32.92 (13.48)	27.83 (13.82)	< .001
K-5	13.79 (5.38)	10.96 (4.80)	.002	12.10 (5.31)	10.67 (4.57)	.011 (unequal variances)	11.71 (5.17)	10.74 (4.68)	.076

CDRS-25 = Resiliency; IES-R = Traumatic Symptoms; K-5 = Distress.

### Qualitative results

As shown in Table 8, most interviewees identified as Qatari (65%), female and married (n = 13, 57%), and with children. Over two third of them (n = 15, 65.2%) have undergraduate education, with age range between 30–40 years old. The majority (n = 19, 82.6%) are employed and have professional occupation.

**Table 8:** Sociodemographic information for the interview sample. Gender, age, education, marital status, occupation, nationality, and number of children by number and percentage of sample.

Demographic Variable	N	%
**Gender:**		
Male	10	43
Female	13	57
**Age**		
30–35	7	30.5
36–40	7	30.5
41–45	5	21.6
46–50	2	8.7
50+	2	8.7
**Education**		
Secondary	4	17.4
Undergraduate	15	65.2
Graduate +	4	17.4
**Marital Status**		
Married	15	65
Divorced/Separated	8	35
**Occupation**		
Private Business	2	8.7
Professional	18	72.3
Unemployed	3	13
**Nationality**		
Qatari	15	65.2
Bahraini	4	17.4
Saudi Arabian	2	8.7
Emirati	2	8.7
**Number of Children**		
1–2	12	52.1
3–4	7	30.5
5–6	4	17.4

#### Negative impacts

There was a great deal of emotional and psychological distress expressed by all 23 interviewees in response to the Blockade. All participants reported significant psychological effects. However, in each case, the severity of the impact varies depending on the extent to which the blockade affected their personal, family, social, and economic lives.

Several emotions were expressed simultaneously: sadness, fear, anxiety, depression, shame, guilt, and anger. Most spoke about the void they felt within themselves due to the blockade. One of the female Qatari Participants:

I can continue my routine life raising my children and living with my husband, but it is all going on with a shade of sadness. Even when we laugh, it does not come out of the heart. Our laughter is no longer real because we feel that within us, something has broken, and when you are broken from the inside, you [I] will not be able to give and feel tired. Every day, I pray to God that this blockade may be finished and that we reunite and come together again (QFP3).

The blockade disrupted people on both ends. Some were ripped off their identities, and others were separated from their families. As a result, there has been a disruption of emotions and feelings of uncertainty. The participant shared her painful experience and struggled to accept reality:

I had contradicting thoughts; I was not able to settle on one place or have a consistent way of thinking; I was not sure about my feelings; I tried to convince myself that it was something temporary, only for some days., but that week, in that same week, I started feeling sick; did I want to leave my children, so I started deluding myself that I was sick? Was I looking for someone to give me more attention and care? I am not sure" (QFP3).

Approximately one-third of respondents reported feelings of depression: A Qatari female reported feeling severe depression because of the blockade’s effects on her living conditions, saying:

I hear voices; I mean, I speak to myself asking: Is this the kind of life you dream of?. . . . I think about my children. All I think about is how to make money to support them, how can I provide for them? Things began to wither around me, and I felt a void of many things in my life … with my husband and our intimate relationship, and with my children and the way I treat them; before I was affectionate, I liked to dress my daughter’s hair and create new styles and things for her. Now it is like, "Quick! Let me tie your hair and go; who is next? Come quickly!" That is the situation (QFP3).

A non-Qatari male participant who continued to live in Qatar after the blockade due to his work conditions talked about the depression he suffered from:

Since the beginning of the blockade, I have gone through a psychological crisis that lasted for two and a half years. Before, I exercised daily, but later I could not continue. After a while, I stopped exercising and gained much weight; I felt emotionally drained. . . It took me two years, during which I didn’t exercise or go running, and I was not motivated to do anything. I had no goals. I tried to escape that situation but relapsed (NQMP1).

In the same context, a Qatari male participant had been separated from his children since the beginning of the blockade. He spoke about his depression and his attempts to overcome it by creating a new family:

Before my second marriage, I was very tired because of the problem with my children, and sometimes I could not sleep. This affected me even at work, even at my place of work. Ultimately, I felt the need not to socialize with anyone. I do not want to talk to anyone. I do not want to go anywhere, either. I felt my mind was busy thinking about them, especially since there was no contact between us (QMP3).

A Qatari female participant reported a severe mental health concern about her daughter using self-harming and non-suicidal self-injurious behaviors to relieve pain: “She [daughter] faced many difficulties and suffered from stress and depression. She began thinking about punishing herself with blades; she did not cut her veins open; instead, she started to cut herself to get pain relief” (QFP5).

Some tried to avoid or deny the stressors when confronted with a highly stressful situation. One of the non-Qatari participants mentioned using ignorance and avoidance:

As for dealing with people, I began to avoid sitting in a place where I could have a conversation related to the crisis. I would avoid sitting with people. I was even a member of WhatsApp groups and left them all. We avoid discussing that issue; we don’t have any conversations about it. Even if someone talks about it, we act as if we haven’t heard anything (NQFP1).

Another Qatari participant reported social isolation after the blockade:

My life circumstances have changed; the circumstances around me have affected me, and my financial status has affected me. My psychological status was terrible because I did not go out or communicate with others. I did not communicate with my friends because of the psychological, financial, and blockade that occurred (QFP3).

#### Emotion-focused coping

*Avoidance*. Some participants maintain peace within their families by avoiding controversial statements that could lead to conflict, as stated by a non-Qatari female participant:

I fear that something wrong has taken place. The thing I fear the most is having a possible argument. I don’t want to upset any family member; I indeed fear that if a bad word is said against Doha, a bad argument might happen, which is unwanted within the family (NQFP5).

Some participants deliberately avoided the blockade and the pressure they were under. A Qatari male participant spoke of his tendency to change his lifestyle and become more involved in social life. He avoided thinking about the problems he was exposed to due to the blockade by excessively shopping. He said:

I started walking outside, moving a lot, and filling my time with doing something else. Then I began to go shopping unbelievably to the extent that I asked myself later why I bought this or that. I found that some things I bought were useless to me (QMP3).

Some participants use avoidance to stop thinking about the new responsibilities they have to face after the blockade. One participant described:

Whenever I think I am responsible and the one in charge, I hate This feeling. I am running away from responsibility. I come late. When anyone talks to me about the issue, I start crying. I no more like to talk about that. Now I don’t even like to go out. I see girls who go out, but I don’t like to spend money but to save money for something more important (QFP6).

Another participant used avoidance strategies to cope with his stress describing:

When I feel weak, I ask my wife to go to her parent’s house, and I take my children to my mother (their grandmother), telling them: ‘I am traveling for 4–5 days, and then I will come back to you.’ This is because I feel weak and cannot bear it. I need 3–4 days to regain my strength. You see it escaping, but it is not; everyone sucks from my blood, my wife, my children from my current wife and ex-wife. How can I get my energy back? I must go away from them all to get my strength and blood back gradually (QMP4).

Some participants refused to discuss their coping strategies with the blockade and considered it a taboo topic, as one of the female participants did: “This topic is problematic for me; I don’t like to talk about it. It is like avoiding discussing all familial, financial, and sexual issues” (QFP6). Another participant stated:

I turned a deaf ear to many people’s words. Sometimes, their words have a negative effect, and I used to be affected in the past. I have become immune to any frustrating words, even from my mother, father, or brother. I got away from negative people. If anyone is negative, I keep away from them. After marriage, I spent most of my time with my wife and parents. We lived in a villa and enjoyed strong family relationships. 90% of my time throughout the month is with the family. I dedicate today to my friends, but it is not at the expense of myself and my family. But I had to rely on myself the most. No one can give me the correct advice or guidance about how to get out of problems. Thank God! (QMP3)

*Venting emotions*. Several participants expressed the accumulation of negative emotions, including patterns of negative thinking:

Will it last for a month, two, three, or four months? When will it end? This feeling is very difficult. I cannot explain it, but while alone, these questions keep bothering me. I don’t tell my husband, children, or even my mum and dad. But it is really hard, especially for someone fond of her parents. I got mentally tired, and I cried sometimes. It is something I didn’t expect (NQFP5).

Using negative thinking as a way to cope with the stress of the blockade left those participants with a devastating pattern that impacted their mood and relationships with significant others. A Qatari woman married to a non-Qatari man used this coping mechanism during the blockade as a way to deal with her husband by stating:

I don’t want to cause problems and worries for myself. I always feel that I suffer from overthinking. Let him [husband] be thankful for my spending on him. I started feeling arrogant. I don’t like reaching this level, but what can I do? I began acting like this out of pressure. I keep reminding him of these things. Sometimes we have arguments, but what can I do? It is not my intention (QFP3).

Another female participant added:

When I sit with my children, I try to forget that they are my children, but when I see them clinging to their father and when he plays with them, I keep silent. I say it is OK, just a temporary hard time that will come to an end. But sometimes, when we argue, I burst right away. Sometimes my attitude is challenging, and I wait for the slightest fault by him to argue with him. Sometimes I feel that I began humiliating him, and I don’t like to reach this level. He started to refrain from asking to get some things that he wanted. He would rather keep silent than say he needs it. He does so not to make me feel that I am humiliating him and that he is spending too much, but still, I have this feeling as something involuntary (QFP6).

*Anxiety and fear*. All participants suffered from anxiety, which first emerged among the female participants, especially those who were married to a non-Qatari spouse. As a result, they express concern regarding their children’s future, education, employment, and access to services. A Qatari male spoke of his fear of depleting Qatari resources, his fear of losing safety, and his inability to protect Qatar without a national army.

We were counting on our neighbors’ ’shield of the island.’ This shield is now a dagger in Qatar. We did not trust each other for a day, so what was the solution? God knows! They brought alliances with international powers [for protection] such as Turkey, China, and Morocco. When do we pay? Our money is gone (QMP5).

A common reaction to the blockade was fear, insecurity, and hopelessness. As one participant described, ’fear’ is the true crisis: “Although we adapted to the situation. We are acclimated and alive. But the fear remained: fear has become the crisis. We can overcome this crisis, but [we fear] will it return? What is going to happen?” (QMP1). Anxiety and fear permeated social relationships as well, in the form of mistrust: “In the last two years after the blockade, one has become cautious in his life with the others, but not paying compliments. You cannot compliment certain people. We don’t complement each other at work and in our social life (NQMP1).

*Blame and guilt*. The participants sometimes blamed themselves for being trapped in this situation. They felt they deserved to be in this position because of actions or circumstances they created for themselves. One Qatari female married to a non-Qatari from the blockading countries blamed herself for her fate, noting: "It was a wrong decision to marry X [non-Qatari man]. We blame each other and lament our fortune, and then we go back to our husbands and families (laughs)” (QFP6).

*Positive reappraisal*. A Qatari male participant expressed his positive feelings about the blockade and how it inspired him to become more involved as a citizen:

I am giving more now, as for participation and volunteering at work. They tried hard to destroy our mental health, but we developed a backlash to this, and as a result, positivity increased at the individual level. It is a very good reaction; all of them plotted against Qatar, so we must protect Qatar and its government. We are right; we are different from them; we have accomplished more; we have reached the global levels not only in sports but also in the institutions of civil society, laws, the constitution that we have …etc. The evidence is the ongoing development and change here. They want us to be dependent on them. However, we are an independent state and have sovereignty. This was a feeling shared by everyone (QMP1).

Another Qatari male participant also expressed positive thoughts and took this path to cope with the overall stressors; he said:

For me, strength is a decision. One can either decide to be happy and accept it or cry, complain, and negatively lose energy. In short, I decided that no one annoys me, a decision I enjoy and live accordingly (QMP5).

#### Problem-focused coping

*Planning*. Several Qatari and non-Qatari female participants coped with the stress of the blockade by planning for secured alternatives to address their problems in case crises arise again, making a better future for themselves and their families. A non-Qatari participant expressed her husband’s desire to buy a house outside Qatar in a country far from the blockade: “My husband is thinking of buying a house abroad, in Oman, for example, considering that Oman is the safest country in the region now (NQFP1). The same participant shared her knowledge of people who have purchased houses outside of Qatar and the Gulf:

It is unbelievable, based on the statistics and the number of people who have bought houses in Turkey since the beginning of the crisis. Each time we meet during a visit, three or four of us women say that they bought houses, in all visits, in every single visit. . . not only by Qataris but by residents too. Residents have bought houses abroad. There is this kind of fear that this region could be exposed to war at any moment, God forbid. May Allah protect the country (NQFP1).

Several other participants also focused on finding alternative citizenship to secure their children’s futures. A Qatari female participant said, “I am thinking about buying them [children] another passport [citizenship]. It is a heavy burden on my shoulders. I used to work and spend on the house needs, but now, things are different. The most important thing is to find a house for them” (QFP1). Another Qatari participant whose children hold non-Qatari citizenship from the blockading countries expressed her desire to invest outside Qatar to obtain another nationality for her children, *“*I am currently trying to obtain a different nationality for my children. I applied to get it through investment; just to get rid of the idea that they are Egyptians” (QFP2).

*Distracting activities and personal growth*. Other participants focused on their health, self-image, and personal growth. A non-Qatari male participant mentioned that he focused on organizing his life priorities, his time with sports, and attending conferences and seminars. He stated:

Now I have more inner peace. I started gathering with people again. I decided that from the beginning of 2020, I would come back to practice sports. Now I am committed. Also, I registered to participate in a marathon. I wouldn’t be committed if I didn’t register my name in such activities. I am also planning to attend lectures and conferences (NQMP1).

Similarly, a Qatari female participant expressed her tendency to focus on her health and appearance to divert her thinking from her problems: “I made a strange decision; I underwent gastrectomy and changed my look and life. I felt I needed a change, so I changed my whole life. Now I’m talking to you after losing about 45–50 kg (QFP2).”

*Religion*. Almost all participants expressed that they have faith in God and that God will have a solution for this crisis. A non-Qatari female participant stated:

The first thing is to believe that Allah, may He be glorified and exalted, will make this crisis temporary. I trust Allah and that He will lift it. We can do nothing but pray during this crisis. We are weak as people (NQFP1).

Another participant said:

I have a strong belief in Allah and am thankful to Him. I know that when Allah loves a servant (worshiper), He will have afflicted him. I always repeat the verse: It is possible to hate something that may be good for you. I spend most of my time supplication and asking Allah for His forgiveness, and when I get up the next day, I am a completely new person who has no problems at all. I always separate work from family so that my performance or personal affairs are not affected. The secret is asking Allah for His forgiveness (NQMP1).

Another Qatari woman added:

My faith in Allah is great, and He is the Generous. God willing, our conditions will get better. So many mothers in Bahrain and Qatar are praying and supplicating to Allah. All the blockade countries, without exception, have been suffering by all means. But what can one do? (NQFP3).

Other participants also emphasized the value of religion and family support:

Firstly, [support] comes from seeking the help of Allah, and secondly, the moral support from my family. This helped me find my strength again. And thirdly, I have priorities in my life: My family comes first, and then my work (QMP2).

*Resiliency*. Although most participants experienced negative impacts due to the blockade, they could identify positive aspects, bouncing back while demonstrating growth and resilience. Some participants showed an ability to make decisions, explain matters, not allow anyone to spread sadness in themselves, and thus be able to resist sad circumstances around them: A Qatari participant mentioned:

The source of power for me is the decision. Do I decide to be happy and accept and be satisfied, or do I slap myself, complain, and pour out my negative energy? In short, my decision has been not to be angered by anyone. That was my decision, and I have lived my life according to it and am enjoying it (QFP3).

Another participant perceived the blockade as an opportunity to reflect and gain a deeper understanding of the conflict. She said:

The positive thing is that my way of thinking has become more profound and more comprehensive. At first, one feels that they do not have exposure, I mean that one’s information is limited. But, after what happened, we got more information and, accordingly, more understanding; we got to know who was right and wrong; we started thinking more profoundly. Now there is dialogue, and we can negotiate and discuss. We have stronger personalities now, and we have really begun. I was not independent, i.e., we always depended on our close relatives (QFP1).

Most participants spoke of their personal growth, character changes, and personality development. One of the Qatari female participants stated:

I have a much stronger personality now than I did before. I have an alternate plan. I have plans for the future. I have a future vision or a life that may have changed more than now; I now feel am stronger. I am pretty in control of my life (QMP5).

The vast majority of the participants expressed an increased awareness about the future and appreciation of their leadership. A male Qatari participant mentioned that Qatari people started to show an increase in their awareness which became a motive for thinking forward and defining plans for the future: One participant said: “They [Qataris] have more awareness about the future. They have started to believe that we must secure our future. We must determine our behavior” (QFP2). Another male participant added: “We have a better awareness of the value of our state, of the treason against our leaders, and more aware that we have to rely on ourselves and our leadership. This was the most crucial point during the blockade” (QMP4).

Independence and self-reliance were among the significant positive changes after the blockade. Most participants stated that Qatar became more productive and dependent on local production, which constituted a protective factor for the state and the citizens. A Qatari participant talked about establishing huge farms that produce high-quality food to be approved in Qatar:

After the blockade, we relied on ourselves; we now have a farm in our country. The quality of the milk and yogurt from our farm is far better than what we used to drink. Even vegetables and fruit are now local production. People are now aware that we can depend on ourselves; we cannot rely on others. This was a knock to awaken us that ‘we’ as Qatari people can work and produce. And as you say, this knock [blockade]was in our favor, we benefited from it. (QMP6).

One participant mentioned:

While the blockade on Qatar devastated some, many others perceived it as an opportunity to become independent and self-sufficient. One of the positive changes is that the country has developed and moved forward… Thank God. It was like a healthy man sleeping well…. Now, it depends on the Qatari youths and has a little movement. A Qatari citizen may have 500,000 Riyals of their own and can use that money to open a shop (QMP7).

Another participant mentioned becoming more involved and engaged in their community: “I am giving more now in participation and volunteering. They [blockading countries] tried hard to destroy our mental health, but we developed a backlash to this, and as a result, positivity increased at the individual engagement level" (QMP1).

On the financial aspect, some participants struggled to hold on to their jobs while others reworked their economic habits, as one participant explains:

Before [the blockade], we used to think of food as something highly important; we don’t buy our needs only; we buy more than what we need. Goods may be unavailable at the market for a long, so we store canned food and the like. Now, we started buying gold. In the past, we had money, but now we are saving our property and buying gold, not Dollars or Riyals. We save in the form of gold. We buy gold bullion bars (QMP1).

Several Qatari participants expressed more appreciation of the new changes they had observed in the job market, as one Qatari participant mentioned:

We had many Gulf nationals in the [Qatari] companies. After the blockade, many people [from the Gulf region] were fired, and many others left. The company had to dismiss many employees, so we got more chances. What were we doing before that? Many people were replacing us for some reason. The company and the country say, "Where have these Qataris been? We have not seen them before!" So, we took a greater chance to take the lead. I felt this is better for us. Previously, the government was blinded and did not notice us. They used to think that Europeans were the best people. No! They now sift and filter them for us to appear (QFP1).

Some participants spoke of work ethics and values, productivity, creativity, good management, and professionalism at the workplace as expressed by one of the participants:

We used to spend five hours at work doing nothing, but now we work eight hours, and we have to submit a daily report on all that we have performed at work to the European manager. It is necessary to come on time, leave on time, and submit a daily work report. I learned how to write a work report. All I used to do in the past was to greet the boss: "Hello Abu Ahmad, how are you? We have done the task you asked us to do (verbally). Now it is not that simple anymore. Everything has become systemized, and all the random tribal work we used to do in Qatar has changed. Work has become more professional. I was amazed. I mean, I started to sit and seek learning from the Europeans., I had to learn about management, reporting systems, and discipline. Today, any Qatari knows more than the Europeans do (QMP5).

In the same context, a Qatari woman described the positive change that occurred to her in her workplace:

There have been many developments at work, and I started to fight more for my rights. In the past, I did not care about that. Then I insisted on getting a promotion, and I got it. I became more interested in my rights (QFP1).

Social and community relations are essential elements of resilience that helps individuals to bounce back and grow through stressful circumstances. Building and strengthening community ties were among the most critical factors kept through interviews. Some participants, especially women, indicated that the blockade was a motivation to increase social and community ties and unite people with each other. One participant mentioned: “this crisis prompted people and society to bond together and work better and increase the sense of nationalism, belonging, and solidarity between people” (QFP5).

Another participant stated:

The whole country, starting with the highest-ranked person to the smallest member of society, has worked on trying to show the value of our country. Even families have become more supportive of each other, bonded more, and settled more. Even the language of dialogue changed. Young adolescent girls and women have not made their conversations trivial or simple. . . No. . . Now they have farsightedness, and now they have more plans for the future (QFP1).

Several participants proudly spoke of the country’s readiness and preparedness to survive any future crisis: A female Qatari participant stated: “One of the resiliency factors was development in all fields in the country. The development raised in health, availability of high-quality equipment, and complete preparation for subsequent crises” (QFP5).

Another participant added:

One of the benefits of the blockade is that we now have seven-star health services instead of five stars. The equipment is better, and we are more aware of the meaning of emergency and crisis. This means that if there is a crisis, Praise be to Allah, it is not a crisis. This is a blockade, and now we are 100% ready for any other thing, whether material or human. Anything that happens, the country is ready. Concerning the health sector, I am sure we are 100% (QFP5).

A non-Qatari participant praised the unity and interconnectedness among the citizens: “The state has changed a lot after the blockade. People have become more coherent…People now have the incentive to work for Qatar. Qatar deserves the best” (NQFP1).

### Discussion

The ABC-X [39] model guided the study to explore and assess the blockade’s psychological distress (X) on Qatari citizens and residents in Qatar. As such, the study focused on the blockade crisis as a traumatic event (A), the available resources (formal and informal support), individual characteristics (coping and resiliency) (B), and their responses to the trauma and perception of the event (C).

### Trauma and stress

The overall survey results showed that the blockade adversely affected Qataris and residents, especially Qatari married women. More specifically, the quantitative results showed that trauma-related stress, especially hypervigilance and intense psychological distress were more prevalent in women than in men and more prevalent among married than single women. Posttraumatic response patterns among women and men with PTSD are consistent with other studies [52,53].

Although women in general show a higher level of vulnerability to stress than men, gender cannot be considered as a single analytical category [54]. In this paper, we argue that gender-specific characteristics of traumatic events may explain these differences rather than responses to them. Using individual characteristics, for example, women in our study exhibited more emotions, were more likely to express their feelings, and seek social support than men. Social norms and gender roles played a major role in shaping these gender differences [55]. Men were more likely to report strength, independence, and superiority, consistent with viewing stress as a sign of weakness, dependency, and vulnerability [56,57]. The results from the survey were supported by the qualitative data. Based on the authors’ observations during individual interviews, both men and women expressed similar and strong emotional reactions to the crisis, but men tended to be embarrassed for showing them. It is also possible to interpret the differences between men and women based on the avoidance as a coping mechanism used more among men as a way of dealing with trauma and as a way of preventing future public humiliations and violations.

Sociocultural and psychological conditioning may impact men and women’s reactions to trauma. For instance, women may be more likely to blame themselves for the trauma and to adopt a more dangerous perception of the world than men [57]. However, the type of trauma experienced by men and women may explain gender differences in trauma reactions more than gender-specific psychological pathways [58,59].

It is important to consider gender inequality when addressing the differences between men’s and women’s responses to trauma. Inequalities based on gender and social exclusion undermine women’s ability to cope and show resilience [60]. Generally, women are socialized more to be relationship-oriented, while men are socialized more to be action-oriented and independent. Thus, women’s socialized roles as caregivers may negatively affect their resilience when faced with stress from family and friends [61]. In the present study, there is a higher risk and vulnerability of traumatic symptoms among women. This is because women in the Middle East cannot grant citizenship to either their husbands or children. Due to the fact that many of the study’s participants were in cross-national marriages, they lacked citizenship rights, which negatively affects their marriages and their ability to access services and support programs (resources). Married women from neighboring countries in the Gulf region were more directly affected by the blockade than men who were married to women from the same countries. Spouses and children suffered as a result of the situation. This trauma had a major impact on the stability of the families and the well-being of their children, especially if the father faced a high risk of deportation. Moreover, studies have shown that cross-national marriages are less stable than marriages between spouses of similar nationalities [2,62], which increases the risk of social exclusion. Furthermore, the social and national identities of women and children were often attributed to distress and anxiety, suggesting that men and women felt differently about citizenship.

### Resilience

The results also showed that men reported a higher level of resilience than women. In general, studies on gender and resiliency report that women usually have lower scores on resilience than men [63–65]. Researchers have explored the relationship between gender roles and resilience. Other studies suggest that women score lower on resilience than men because resilience measures fail to capture gender roles, social expectations, perceptions, and environmental factors that impact resilience outcomes [66]. The cultural constructs that maintain certain ideals of gender and gender identity, along with the gender-based citizenship status of Qatari women, contributed to the vulnerability of women [2]. Gendered citizenship appears to have exacerbated psychological distress in Qatar’s cross-national families. Their perceptions involve recognition, the acknowledgment of women as equal partners, equal rights, visibility, inclusion, engagement, and access to material benefits. The loss of citizenship may have caused the participants to feel rejected, alienated, excluded, and marginalized.

Despite high levels of stressors, most participants reported a high level of resilience, with men reporting higher resilience than women. The quantitative results found that resiliency helped alleviate general distress but not symptoms following a significant traumatic event. Qualitative findings also indicate a high degree of resilience at the micro, mezzo, and macro levels. Most participants used problem-based coping strategies, which led to a high level of resilience. Although coping is not the same as resilience, studies show a close association between the two [67,68]. Essentially, resilience determines which coping strategies individuals use, and how effectively they cope with stress affects their resilience. A study looking at differences in resilience and coping strategies between male and female Holocaust survivors found the use of problem-focused and emotion-focused coping strategies were equally used by women, while most male participants relied on problem-focused strategies [69]. The same study found that male participants experienced constant fear, but they expressed significantly greater confidence in surviving their circumstances [69]. Men reported taking control of their situations while women attributed their survival to others. Other studies also show that women are more likely than men to use self-blame and denial as coping strategies [70–72]. Women may also rely more on strategies such as rumination [73,74], which can contribute to the onset of depressive episodes and interact with other negative cognitive styles to prolong their duration, especially in women [73–77].

Men tend to report higher levels of mastery, which correlates to lower levels of anxiety [78]. Social factors also influence self-acceptance and environmental mastery, the two measures underlying the heritability of resilience [79]. Neither of these measures showed to be differentially heritable between men or women, though the authors suggest that men may have a greater opportunity to express and be rewarded for expressing signs of resiliency [79]. It was also found that females exhibited greater resiliency gains than males in a study about the effects of an experiential, adventure-based program on fifth-grade Latino students’ resilience [80]. This study provides a similar explanation regarding the female adventure education experience. It also suggests that cultural norms like machismo may play a role in males not being willing to risk failure.

Based on the literature, during a political crisis, resiliency may take a different form and practice. In Palestine, for example, resilience is connected to nonviolent resistance (sumud) [81]. A Palestinian sumud is a nonviolent resistance practice based on historical legends and national or religious beliefs, especially during wartime [82]. Women react differently to chronic political violence than men [83]. They perceive circumstances as threatening and less manageable, and they experience more distress when faced with chronic political violence.

Furthermore, women who have experienced problems due to the blockade tend to seek more support. In contrast to other studies [84,85], support indicators (formal and informal) were not associated with resilience among Qatari women. They did not alleviate their stressors, nor did it make them more resilient. It may be possible to connect the type of support they sought to receive, which in this study was their goal to pass their citizenship to their children and spouses rather than simply have access to social and health care services [11]. According to the findings, factors such as the fear of deportation and uncertainty regarding their families’ future can lead to higher stress, PTSD, and a lower level of resilience.

### National resilience

While there were differences between men and women, Qatari and non-Qatari women, and married and single women, the qualitative and quantitative data demonstrate that Qatari citizens and residents show a high level of resilience. Qataris became more involved in their political situation, engaged in volunteer work, became more self-reliant, appreciated their leaders, and became more attached to their Qatari identity.

At the macro level, Qatar became more economically independent and robust while driving it further away from the GCC. With most of its consumption coming from imports, Qatar has become more independent by creating alternative supplies while expressing heightened sentiments on social media [86]. Positive emotions were used as coping strategies to guide residents and the country toward resilience [30]. These coping strategies have also enhanced national identity, pride, and the will to cope with uncertainty [86,87]. The resilience and strength of Qataris can also be attributed to two main factors. Qatar’s resilience is due to its vast wealth and strong leadership under Sheikh Tamim, who uses servant leadership techniques [88] manifested by his creativity and commitment to national resilience. During the blockade, to help manage the stressors following the event, Qatari leadership used a ‘servant leadership’ approach that prioritized citizens’ needs, interests, and concerns, as well as the concerns of others within the larger community and country as a whole [89]. This approach can enhance resilience among citizens by fostering a sense of community and collective purpose and promoting open and effective communication. Servant leaders tend to empower and support their followers, which can help individuals and groups develop the skills, confidence, and resources they need to navigate difficult situations and bounce back from adversity.

### Practice and policy implication

This research study is a significant milestone in the mental health research specific to Qatar under this particular political climate. The paper provides a preliminary assessment of the specific experiences and challenges faced by Qatari citizens and residents’ families. In addition, the mixed-methods results provide a better understanding of the psychological effects of the blockade on the well-being of Qatari families, their perceived support, and their coping strategies. At the same time, the results identified challenges and limitations that prevent Qatari families, especially in cross-national marriages, from receiving timely support and services before these become risk factors for various mental health issues that may disrupt their social, psychological, financial, and familial situation. The present findings can inform social work practice, research, and advocacy at the individual, family, community, societal, and governmental levels.

#### Individuals and families

The focus on individuals’ well-being, especially of women and children, is consistent with the goals of mental health research to generate knowledge about the most vulnerable and underserved populations. Identifying and addressing the mental health needs, support, coping strategies, resiliency, and growth among affected families will help providers improve the quality of care, build their resiliency, and strengthen their problem-focused coping strategies. The expected growth will also enable them to thrive and increase their functioning. Parents’ mental distress will likely impact their children’s well-being [90]. By taking care of the entire family’s mental health, each member will be healthier and more effective, reducing the risk of mental illness and violence [91].

Future studies should pay more attention to children’s perceptions of this event, especially among those who have experienced separation from their families and communities. New interventions must provide a secure environment in which children can express their anxieties, fears, anger, sadness, emotions, and negative thoughts. Moreover, children and parents of cross-national families can benefit from joint or separate intervention programs to ease tension and trauma. Studying local variations and universal themes in diverse contexts related to this target group would be a great initiative for this suggested intervention. The results of this study may invite others to replicate similar efforts to deepen and extend the advancement of mental health providers with families with similar experiences.

#### Community and societal

These findings will lay the ground for social and clinical intervention trials that help mental health providers develop more effective services for the Qatari families directly affected by the blockade. Social workers and mental health providers will better understand their target populations. They will be able to advocate for reforms that could reduce some of the stressors that affect parents’ coping mechanisms while highlighting their strengths and resilience. Findings can be developed into therapeutic tools and service interventions that guide mental health providers in planning culturally sensitive services, which take a strength-based approach highlighting the individual and community resources, protective factors, and resiliency.

This study brings new insights into what, when, who, and how to serve this population. It is important to engage mental health providers and social workers in addressing these challenges through evidence-based and core competencies of various interprofessional at Qatari medical and mental health centers. The results will also encourage the Qatari society to appreciate the social workers’ responsibilities in the helping process, especially when addressing traumatized families.

#### Governmental

The collaboration between policymakers and mental health care providers is necessary to enhance mental and physical health resilience and promote security and health in the Middle East [92]. Interventions should promote coping strategies, resilience, life skills, and symptom management. Mental health professionals should also offer trauma-focused cognitive behavioral therapy, narrative exposure, and child-centered therapy.

In the long term, the study results provide new perspectives and insights on how to increase the number of quality resources and services that can help increase this population’s levels of coping and resilience. In turn, future intervention and policy models can be used to develop preventive screening tools and culturally responsive and effective treatments and services for distressed families. Suggesting new preventive services can also help parents improve their family members’ quality of life and overall social and psychological well-being, particularly when they become aware of their strengths and available resources.

This study contributes to emerging literature investigating how the political blockade affects the health and well-being of Qatari citizens and residents, their families, and children. Study findings will inform policymakers about the types of stress and the challenges experienced by these individuals during a political crisis like the Blockade. It is essential that policymakers identify the source of stressors associated with a lack of citizenship, especially among children of Qatari women, and develop new resources to assist cross-national families in building their resilience during difficult times. Most resilience programs are designed to meet the needs of men and may not address women’s specific issues, such as gender-based citizenship. Programs should consider women’s and men’s different needs and experiences. Therefore, policies that promote resilience must ensure that both men and women have equal access to the same opportunities, citizenship rights, and resources necessary for resilience building.

To conclude, it is crucial to examine whether political conflict affects individuals’ mental health and psychological well-being and how people’s social environments, individual characteristics, resources, and leadership approaches may provide them with protection and resiliency.

## Limitations

For the quantitative part, the study used a cross-sectional design of a convenience sample, which limited the possibility of drawing rigorous conclusions about the causes and consequences of stress and resilience. In the qualitative component of the study, only participants in cross-national families were included, which limits comparisons with other families and residents of Qatar. Although measures and scales used in this study were translated and validated in Arabic, cultural differences could affect their reliability and validity, especially when reactions to traumatic events are strongly stigmatized. In addition, perceived gender appropriateness may affect survey responses [93,94]. Generally, socially desirable responses have been identified as a problem in quantitative research and should continue to be considered. We suggest incorporating gender sensitivity into resilience characterizations and measurements to promote both genders’ well-being. Identifying gender-based differences can be used to tailor interventions and support programs specifically designed to address the needs of each gender. By developing gender-sensitive indicators, we can comprehensively understand resilience in different populations.

## Supporting information

S1 File Total data set. This is the total data set for the research.
(XLSX)
